# Gallium Trichloride Fluid: Dimer Dissociation Mechanism, Local Structure, and Atomic Dynamics

**DOI:** 10.3390/molecules29061358

**Published:** 2024-03-19

**Authors:** Maxim Khomenko, Anton Sokolov, Andrey Tverjanovich, Maria Bokova, Mohammad Kassem, Takeshi Usuki, Eugene Bychkov

**Affiliations:** 1 National Research Centre, Kurchatov Institute, Shatura, Moscow 140700, Russia; khomenkolaser@gmail.com; 2 Laboratory of Biophotonics, Tomsk State University, Tomsk 634050, Russia; 3 Laboratoire de Physico-Chimie de L’atmosphère, Université du Littoral Côte d’Opale, 59140 Dunkerque, France; anton.sokolov@univ-littoral.fr (A.S.); maria.bokova@univ-littoral.fr (M.B.); mohamad.kassem@univ-littoral.fr (M.K.); 4 Institute of Chemistry, St. Petersburg State University, St. Petersburg 198504, Russia; andr.tver@yahoo.com; 5 Faculty of Science, Yamagata University, Yamagata 990-8560, Japan; usuki@kdw.kj.yamagata-u.ac.jp

**Keywords:** supercritical gallium trichloride, dimer dissociation mechanism, first-principles molecular dynamics

## Abstract

Molten gallium trichloride emerges as a promising solvent for oxidative metal recycling. The use of supercritical fluid enhances the performance and kinetics of metal dissolution due to significantly lower viscosity in the reaction media. Additionally, the dual molecular nature of gallium trichloride, existing as edge-sharing ES-Ga_2_Cl_6_ dimers at low temperatures and high pressure, or flat trigonal GaCl_3_ monomers in the vicinity of the critical point and low pressures, creates the possibility to tailor the chemical geometry to a particular metallic species. Nevertheless, the mechanism of dimer dissociation, local structure, and atomic dynamics in supercritical gallium trichloride fluids are not known. Using first-principles molecular dynamics, validated by comparison with our high-energy X-ray diffraction results, we illustrate the elementary steps in dimer dissociation. These include the formation of intermediate corner-sharing CS-Ga_2_Cl_6_ dimers, the partial disproportionation of GaCl_3_ monomers at high temperatures and low pressures, changes in the local environment of molecular entities, and unusual atomic dynamics in supercritical fluids.

## 1. Introduction

**Figure 1:**
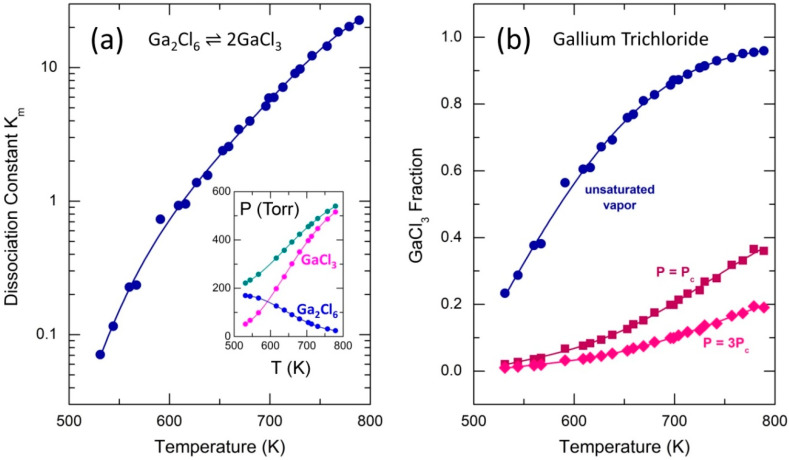
Calculating the parameters of the dissociation reaction Ga_2_Cl_6_ ⇌ 2GaCl_3_ using the reported total and partial vapor pressures of Ga_2_Cl_6_ and GaCl_3_ [16]: (**a**) the dissociation constant $K_{m}\left(T\right)$ as a function of temperature and (**b**) the molar fraction $x_{{\mathrm{GaCl}}_{3}}\left(T\right)$ as a function of temperature under different pressure conditions. The insert in (**a**) represents one of the pressure measurement experiments [16]. The calculation details are given in the Supporting Information.

Molten gallium trichloride appears to be a promising oxidative solvent for the recycling of metals, including rare earth elements, platinum and transuranium group metals, and heavy pnictogens and chalcogens [1,2,3,4,5,6,7]. The use of supercritical fluid additionally yields a much lower viscosity in the reaction media, higher diffusion coefficients, and favorable kinetics. Supercritical solvents, particularly carbon dioxide fluid, have already been successfully used for the oxidative dissolution of copper and zinc in the microelectronic industry (see, for example, Ref. [7] and references therein). Supercritical CO_2_ possesses high surface wetting, extremely low viscosity, low surface tension, and a low dielectric constant, which are beneficial for metal recycling. The dual molecular form of gallium trichloride, existing as edge-sharing tetrahedral dimers ES-Ga_2_Cl_6_ or flat trigonal GaCl_3_ monomers, creates the possibility to adjust the chemical geometry to a particular metal species. This is achieved by choosing either a tetrahedral chlorinated agent [1,2] or a planar molecular shape with an easily accessible gallium counterpart, facilitating the creation of a metal–gallium bond [3]. The dimer dissociation, Ga_2_Cl_6_ ⇌ 2GaCl_3_, basically depends on temperature and pressure [8,9]. Unsaturated gallium trichloride vapor primarily transforms from its dimeric form at low temperatures to a monomeric gas above 700 K (see the inset in Figure 1a). However, the monomeric fraction $x_{{\mathrm{GaCl}}_{3}}$ decreases with increasing pressure and appears to be $x_{{\mathrm{GaCl}}_{3}}$ ≈ 0.20 at the critical temperature $T_{c}$ = 694 K and pressure $P_{c}$ = 6.11 MPa [10], Figure 1b. Further increasing the pressure ($P>P_{c}$) leads to a monotonic decrease in $x_{{\mathrm{GaCl}}_{3}}$. These trends are supported by Raman and diffraction studies of unsaturated vapors and experiments conducted under high-pressure conditions [11,12,13,14]. In addition, a small fraction of Ga_3_Cl_9_ trimers (2–3%) was found in a low-temperature vapor, disappearing above 600 K [15].

Clear thermodynamic results do not unveil the exact microscopic mechanism of the dimer dissociation on an atomic scale. We have used first-principles molecular dynamics (FPMD) above the critical point to identify the elementary steps of the dissociation reaction and associated structural changes. The quality of FPMD modeling was validated using high-energy X-ray diffraction results of normal gallium trichloride liquid and supercritical fluid, reported elsewhere [14].

## 2. Results and Discussion

### 2.1. Validation of the FPMD Modeling by High-Energy X-ray Diffraction

**Figure 2:**
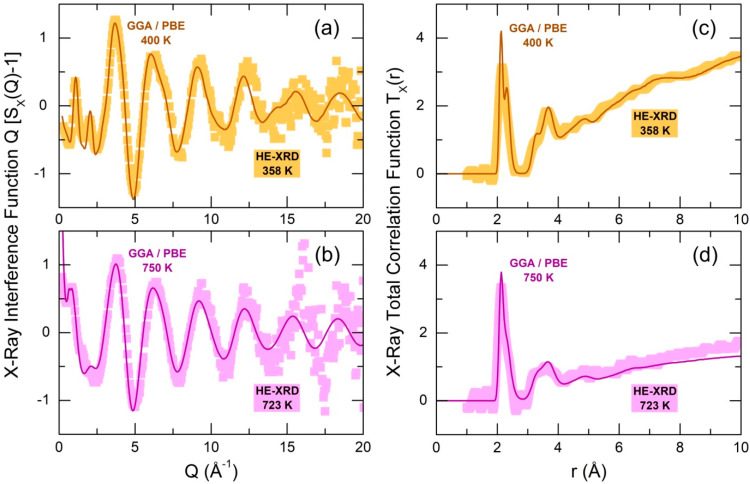
FPMD-derived X-ray interference functions $Q[S_{X}\left(Q\right)-1]$ for molten GaCl_3_ at (**a**) 400 K and (**b**) 750 K, compared to experimental data at comparable temperatures; FPMD and experimental X-ray total correlation functions $T_{X}\left(r\right)$ at (**c**) 400 K and (**d**) 750 K. The solid lines represent FPMD results, and the solid squares denote experimental data. The difference in the $T_{X}\left(r\right)$ level at higher r between the experimental and FPMD results is caused by variations in number density, with a significantly smaller value at 750 K (0.00902 atoms Å^−3^) compared to 723 K (0.013 atoms Å^−3^).

The FPMD modeling, employing the general gradient approximation and PBE exchange–correlation functional (GGA/PBE), used a simulation box of 800 atoms (200 Ga + 600 Cl). The box size was chosen to match the experimental number density. The simulations reveal good agreement with HE-XRD results below and above the critical point, Figure 2. In supercritical gallium trichloride fluid, a strong small-angle X-ray scattering (SAXS) is observed, attributed to mesoscopic voids and cavities. Despite the insufficient size of the FPMD box, a distinct SAXS signature of mesoscopic voids is clearly reproduced in Q-space, emphasizing the high quality of the first-principles simulations.

The real-space functions mimic asymmetric Ga-Cl nearest-neighbors (NNs), which are related to terminal Ga-Cl(t) and bridging Ga-Cl(b) contributions in ES-Ga_2_Cl_6_ dimers at 2.12 and 2.31 Å, respectively. These two populations are rather well resolved in normal liquids but overlap in supercritical fluids. The multimodal second neighbor features include Ga-Ga, multiple Cl(b)-Cl(b), Cl(b)-Cl(t), and Cl(t)-Cl(t) correlations between 3 and 4 Å, as well as more distant Ga-Cl(t) second neighbor contacts at 5 Å. A broad unresolved feature, roughly centered at approximately 7 Å, represents a center–center correlation distance between Ga_2_Cl_6_ dimers. This separation is easily distinguished in normal liquids but becomes flat and essentially invisible in supercritical fluids.

### 2.2. Dimer Dissociation in Supercritical Fluid

**Figure 3:**
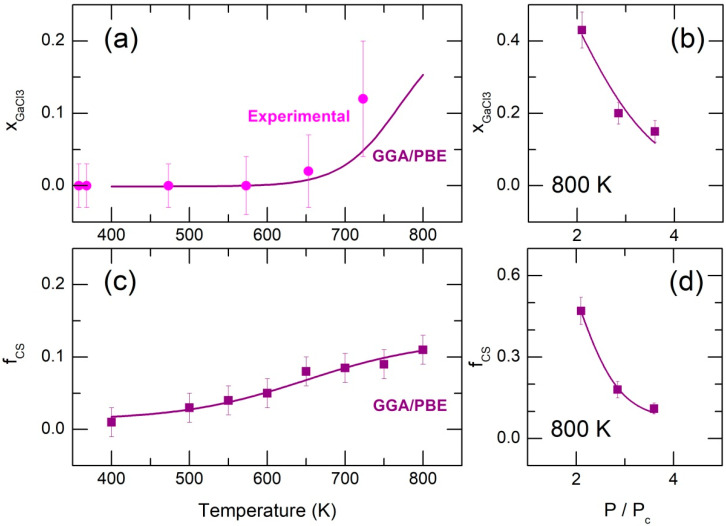
Experimental (magenta) and FPMD-derived (purple) monomeric fraction $x_{{\mathrm{GaCl}}_{3}}$ in liquid and supercritical gallium trichloride as a function of (**a**) temperature, and (**b**) relative pressure $P/P_{c}$; population of CS-Ga_2_Cl_6_ dimers $f_{\mathrm{CS}}$ as a function of (**c**) temperature, and (**d**) relative pressure $P/P_{c}$.

Our HE-XRD measurements were carried out under high-pressure conditions due to the small volume of the sealed silica tube containing gallium trichloride. At the critical temperature $T_{c}$ = 694 K and above, the estimated pressure P was significantly higher than the critical pressure $P_{c}$, with $P/P_{c}$ ≳ 3.6. The experimental and FPMD-derived monomeric fractions ($x_{{\mathrm{GaCl}}_{3}}^{\exp}$ ≈ 0.12 at 723 K and $x_{{\mathrm{GaCl}}_{3}}^{\mathrm{FPMD}}$ = 0.15 at 800 K, Figure 3a) are reasonably consistent with $P/P_{c}$ and the thermodynamic approach, as shown in Figure 1b.

Further increase in $x_{{\mathrm{GaCl}}_{3}}$ at 800 K was achieved by enlarging the simulation box, thereby decreasing the pressure to $P/P_{c}$ = 2.85 and 2.1. The reduced pressure raised the monomeric fraction by nearly a factor of three, reaching $x_{{\mathrm{GaCl}}_{3}}^{\mathrm{FPMD}}$ = 0.43 at $P/P_{c}$ = 2.1 (Figure 3b). In addition, an approximate extrapolation to $P/P_{c}$ = 0 yields $x_{{\mathrm{GaCl}}_{3}}^{\mathrm{FPMD}}$ ≈ 1.0. These results appear consistent with the thermodynamic model, which predicts $x_{{\mathrm{GaCl}}_{3}}$ = 0.96 for unsaturated vapor at 800 K.

**Figure 4:**
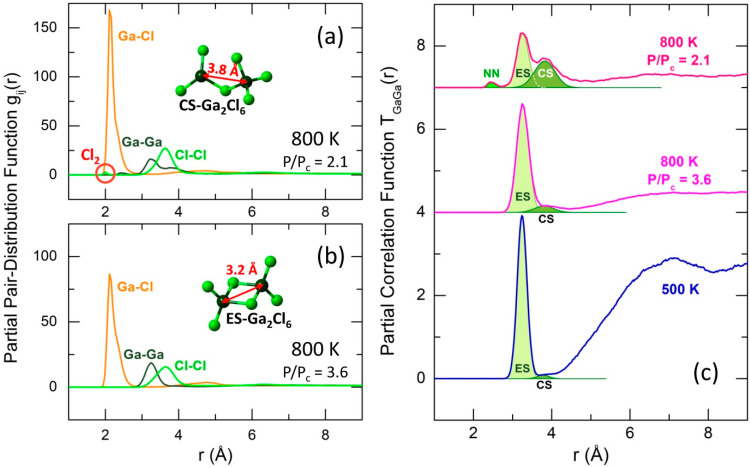
FPMD-derived partial pair-distribution functions $g_{\mathrm{ij}}\left(r\right)$ at (**a**) 800 K ($P/P_{c}$ = 2.1) and (**b**) 800 K ($P/P_{c}$ = 3.6); (**c**) fitting the Ga-Ga partial correlation function $T_{\mathrm{GaGa}}\left(r\right)$ at different temperatures and pressures; short Ga-Ga second neighbor contacts at 3.2 Å correspond to edge-sharing ES-Ga_2_Cl_6_ dimers (the insert in (**b**)), and long Ga-Ga contacts at 3.8 Å correspond to corner-sharing CS-Ga_2_Cl_6_ dimers (the insert in (**a**)). The Cl-Cl and Ga-Ga nearest neighbors (NNs) at 2.0 and 2.4 Å, respectively, indicate a partial disproportionation of GaCl_3_ monomers (GaCl_3_ ⇌ GaCl + Cl_2_) above the critical temperature and at lower pressure ($P/P_{c}$ = 2.1).

Moreover, the partial pair-distribution functions $g_{\mathrm{ij}}\left(r\right)$ at 800 K and reduced pressure ($P/P_{c}$ = 2.1) show particularly interesting results (Figure 4a). The Ga-Ga partial function $g_{\mathrm{GaGa}}\left(r\right)$ at higher pressure and lower temperatures typically reveals a single predominant peak at 3.2 Å, corresponding to short Ga-Ga second neighbor distances in ES-dimers, see the insert in Figure 4b. This single peak transforms into a bimodal feature with decreasing $P/P_{c}$. The high-r counterpart at 3.8 Å indicates a significant fraction of corner-sharing CS-Ga_2_Cl_6_ dimers (the insert in Figure 4a), which are formed after breaking one Ga-Cl(b) bond in ES-dimers. The CS-entities seem to be intermediate varieties in the ES-dimer dissociation process.

However, a close inspection of $T_{\mathrm{GaGa}}\left(r\right)$ partials over the available T- and P-ranges shows that a small population of CS-Ga_2_Cl_6_ also exists at lower temperatures and higher pressures. The $T_{\mathrm{GaGa}}\left(r\right)$ fitting (Figure 4c) allows for a quantitative analysis of the CS-population. The fitting results, shown in Figure 3c, reveal a weak temperature dependence of the CS-population $f_{\mathrm{CS}}$ at high $P/P_{c}$ and a sudden rise in $f_{\mathrm{CS}}$ at reduced pressure (Figure 3d). The observed similarity between $x_{{\mathrm{GaCl}}_{3}}$ and $f_{\mathrm{CS}}$ suggests that the transformation ES-Ga_2_Cl_6_ → CS-Ga_2_Cl_6_ is a first elementary step in the dimer dissociation process.

As a result of dissociation, the shape of the asymmetric Ga-Cl NN peak changes with increasing temperature and reducing pressure. The fraction of Ga-Cl(t) pairs at 2.12 Å increases, while the population of Ga-Cl(b) at 2.31 Å decreases.

**Figure 5:**
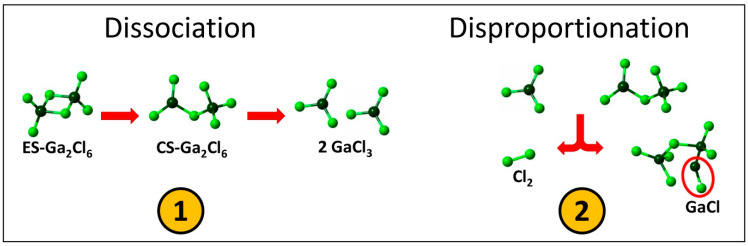
Schematics of ① ES-Ga_2_Cl_6_ dimer dissociation and ② GaCl_3_ monomer disproportionation.

We should also note the emergence of a small but distinct fraction of Ga-Ga (2.45 Å) and Cl-Cl homopolar bonds (2.01 Å) at 800 K and $P/P_{c}$ = 2.1, Figure 4a. The Cl-Cl bonds are associated with molecular chlorine formed in the supercritical fluid resulting from the monomer disproportionation: GaCl_3_ ⇌ GaCl + Cl_2_. Gallium monochloride is typically observed at high temperatures, becoming predominant above 1100 K [17,18]. In our case, GaCl remains elusive, either forming intermediate species via homopolar Ga-Ga and heteropolar Ga-Cl bonding or persisting as diatomic molecules (with a residual 50% occurrence in the latter two cases). Schematics of the dissociation and disproportionation reactions are summarized in Figure 5.

### 2.3. Local Geometry of Tetrahedral and Trigonal Units

**Figure 6:**
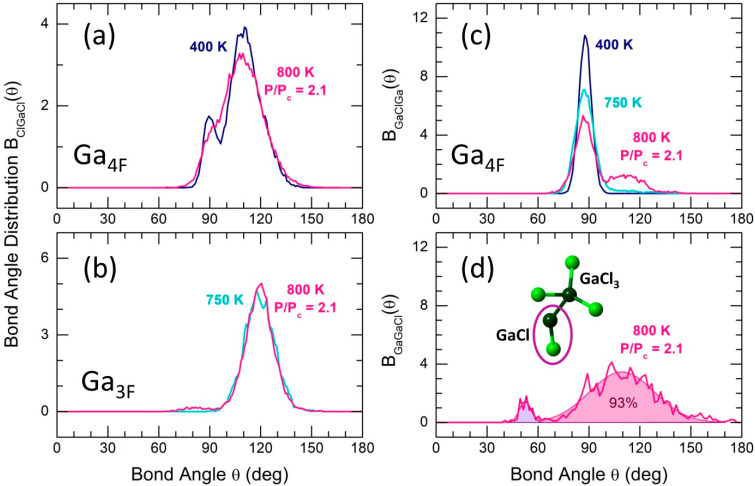
FPMD-derived bond angle distributions: $B_{\mathrm{ClGaCl}}\left(\theta \right)$ for (**a**) four-fold Ga_4F_ and (**b**) three-fold Ga_3F_ coordinated gallium species at different temperatures; (**c**) $B_{\mathrm{GaClGa}}\left(\theta \right)$ for Ga_4F_ atoms at 400, 750, and 800 K; and (**d**) $B_{\mathrm{GaGaCl}}\left(\theta \right)$ at 800 K ($P/P_{c}$ = 2.1). The insert in d shows a transient species GaCl_3_ + GaCl. See the text for further details.

The local geometry of tetrahedral dimers depends on temperature and pressure. Figure 6a reveals the contrasting Cl-Ga-Cl bond angle distributions $B_{\mathrm{ClGaCl}}\left(\theta \right)$ for normal gallium trichloride liquid at 400 K and supercritical fluid at 800 K with reduced $P/P_{c}$. The $B_{\mathrm{ClGaCl}}\left(\theta \right)$ function at 400 K exhibits a well-resolved, slightly asymmetric bimodal distribution centered at 89° and 111°. The tetrahedral angular contribution is related to Cl-Ga-Cl triplets involving terminal Cl(t) species, with a low-θ minority corresponding to a nearly square ring of the ES-dimer. The Ga-Cl(b)-Ga counterpart of the ring, $B_{\mathrm{GaClGa}}\left(\theta \right)$, is centered at 88° (Figure 6c), reflecting its folded non-planar nature. In contrast, the bimodal $B_{\mathrm{ClGaCl}}\left(\theta \right)$ distribution for remaining tetrahedra in the supercritical fluid at reduced pressure is poorly resolved, with a significantly diminished low-θ angular part of the central Ga-Cl(b)-Ga-Cl(b) ring due to a partial ES-CS conversion. Additionally, the tetrahedra become less distorted, with the major tetrahedral contribution centered at 109°. The ES-CS conversion becomes clearly illustrated by the $B_{\mathrm{GaClGa}}\left(\theta \right)$ function at 800 K and reduced pressure (Figure 6c). A previously symmetric $B_{\mathrm{GaClGa}}\left(\theta \right)$ transforms into a bimodal angular distribution. The emerging high-θ angular population centered at 112° is associated with CS-connectivity in partially transformed Ga_2_Cl_6_ dimers.

The trigonal GaCl_3_ units are primarily flat and possess approximate $D_{3h}$ symmetry. The broadened $B_{\mathrm{ClGaCl}}\left(\theta \right)$ function is centered at 119° and essentially remains intact as a function of temperature and pressure (Figure 6b). Nevertheless, a weak population (≈4%) of distorted trigonal units appears at 800 K and reduced pressure, revealing a low-θ component centered at 87 ± 4°.

The geometry of GaCl diatomic entities, interacting with trigonal monomers or CS-dimers through the formation of Ga-Ga homopolar bonds (see Figure 5 and the insert in Figure 6d), is characterized by Ga-Ga-Cl bond angles. The $B_{\mathrm{GaGaCl}}\left(\theta \right)$ distribution is essentially centered at 109° (Figure 6d), indicating a tetrahedral geometry of the arising transient units. Additionally, there is a small population of highly distorted species with $B_{\mathrm{GaGaCl}}\left(\theta \right)$ peaked at 52°.

Complementary information on local geometry yields the orientation order parameter q [19,20]:

(1) $$q=1-\frac{3}{8}\sum_{j=1}^{n-1}\sum_{k=j+1}^{n}{\left(\cos\psi_{jk}+\frac{1}{3}\right)}^{2},$$

where $\psi_{jk}$ is the Cl-Ga-Cl angle of a tetrahedral (n = 4) or trigonal (n = 3) ${\mathrm{GaCl}}_{n}$ unit. The average value of q changes between 0 for an ideal gas and q = 1 for a regular tetrahedral network.

**Figure 7:**
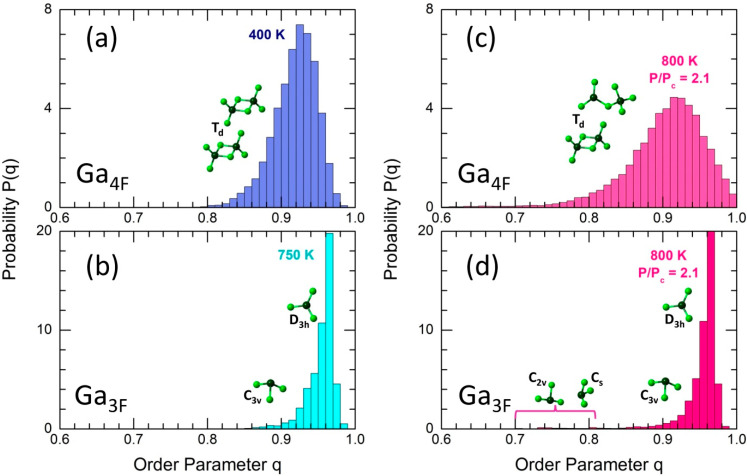
Orientational order parameter q for 4-fold Ga_4F_ and 3-fold Ga_3F_ coordinated Ga-Cl entities: (**a**) Ga_4F_ at 400 K, (**b**) Ga_3F_ at 750 K, (**c**) Ga_4F_ at 800 K ($P/P_{c}$ = 2.1), and (**d**) Ga_3F_ at 800 K ($P/P_{c}$ = 2.1). The inserts show typical Ga-Cl units of different symmetry. See the text for further details.

The GaCl_4_ tetrahedra are strongly distorted in ES-Ga_2_Cl_6_ dimers, revealing two different Ga-Cl(t) and Ga-Cl(b) bond lengths and a bimodal $B_{\mathrm{ClGaCl}}\left(\theta \right)$ angular distribution. As a consequence, the order parameter q exhibits an asymmetric P(q) probability function, centered at q = 0.93 (Figure 7a). Nevertheless, a non-tetrahedral geometry at q < 0.8 [21,22] is basically missing, as well as regular tetrahedral units with q ≈ 1. The P(q) tetrahedral function in supercritical fluid at reduced pressure becomes strongly broadened; however, the maximum is slightly shifted to a higher q, and the high-q tail extends up to q = 1. On the other hand, a larger fraction of regular tetrahedra is compensated by a higher population of strongly distorted entities and even non-tetrahedral species (4.6%).

The shape of the trigonal P(q) function, peaked at q = 0.97, remains essentially intact in supercritical fluids (Figure 7b,d), indicating the relative stability of the trigonal geometry. GaCl_3_ monomers of $D_{3h}$ symmetry are consistent with this maximum. Symmetric (umbrella-type) or asymmetric bending temporarily alters the Cl-Ga-Cl bond angles, reducing the symmetry to $C_{3v}$ or lower, mostly explaining the P(q) shape. However, a small fraction (2.3%) of highly distorted pyramids with q ranging between 0.70 ≤ q ≤ 0.82 exists at 800 K and reduced pressure. Considering the small population of Cl-Ga-Cl angles at about 90° (Figure 6b), these GaCl_3_ units could have $C_{s}$ symmetry, including T-shaped planar entities ($C_{2v}$, q = ¾), as shown in the insert of Figure 7d.

### 2.4. Dynamics in Supercritical Fluid

The much lower viscosity of supercritical fluids compared to normal liquids significantly accelerates the rate of chemical processes involving supercritical solvent [7,23], emphasizing the importance of understanding atomic dynamics in supercritical gallium trichloride for practical applications. The mean-square displacements (MSD) of Ga and Cl, $\langle r_{i}^{2}\left(t\right)\rangle$, were utilized for diffusion calculations.

(2) $$\langle r_{i}^{2}\left(t\right)\rangle =\langle \frac{1}{N_{i}}\left\{\sum_{i=1}^{N_{i}}{\left[r_{i}\left(t\right)-r_{i}\left(0\right)\right]}^{2}\right\}\rangle ,$$

where $r_{i}\left(t\right)$ and $r_{i}\left(0\right)$ are the positions of particle i at time t and the initial time, respectively; $N_{i}$ represents the total number of particles in the simulation box, and the angle brackets denote the average over initial times.

**Figure 8:**
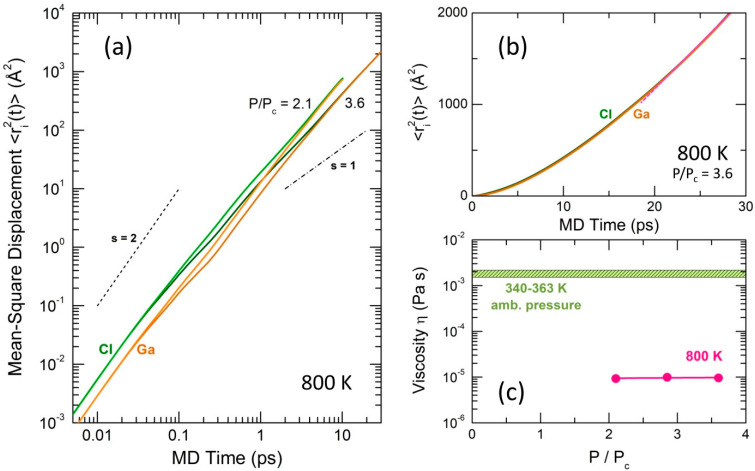
Gallium $\langle r_{Ga}^{2}\left(t\right)\rangle$ and chlorine $\langle r_{\mathrm{Cl}}^{2}(t)\rangle$ mean-square displacements on (**a**) log–log and (**b**) linear scales; (**c**) experimental GaCl_3_ viscosity η(T,P) at ambient pressure between 340 and 363 K [25], and DFT-derived viscosity at 800 K and different pressure (2.1 ≤ $P/P_{c}$ ≤ 3.6) (this work). See the text for further details.

Typical gallium and chlorine MSD in supercritical fluid at 800 K and different pressure ($P/P_{c}$ = 2.1 and 3.6) are shown in Figure 8a on a log–log scale. They differ significantly from the MSDs for normal liquid (Figure S1 in the Supporting Information), which exhibit two distinct regimes: (i) below 30 fs and (ii) above 1 ps. The ballistic regime (i) is characterized by a power-law dependence, $\langle r_{i}^{2}\left(t\right)\rangle \propto t^{s}$, with the power-law exponent s = 2 [24]. The diffusion regime (ii) reveals a linear dependence of $\langle r_{i}^{2}\left(t\right)\rangle$ as a function of time t, that is, s = 1. These two regimes are schematically shown in Figure 8a. In contrast, the supercritical fluid does not exhibit the diffusion regime. Instead, the slope s decreases slightly above 1 ps but remains considerably higher than s = 1.

The gallium $D_{\mathrm{Ga}}$ and chlorine $D_{\mathrm{Cl}}$ diffusion coefficients were derived from $\langle r_{\mathrm{Ga}}^{2}\left(t\right)\rangle$ and $\langle r_{\mathrm{Cl}}^{2}\left(t\right)\rangle$ using the Einstein relation.

(3) $$D_{i}=\frac{1}{6}\underset{t\to \infty }{\lim}\frac{\partial \langle r_{i}^{2}\left(t\right)\rangle }{\partial t}.$$

While the supercritical fluid does not exhibit the diffusion regime, the $\langle r_{i}^{2}\left(t\right)\rangle$ function is not linear on a linear scale and displays an upward curvature. Therefore, the derived diffusion coefficients are only approximate.

The effective diffusion coefficient was defined as $D_{\mathrm{eff}}\left(T,P\right)=\frac{1}{4}D_{\mathrm{Ga}}\left(T,P\right)+\frac{3}{4}D_{\mathrm{Cl}}\left(T,P\right)$ and used for viscosity η(T,P) calculation applying the Stokes–Einstein relation.

(4) $$\eta \left(T,P\right)=\frac{k_{B}T}{6\pi D_{\mathrm{eff}}\left(T,P\right)r_{H}},$$

where $r_{H}$ is the effective hydrodynamic radius, and $k_{B}$ and T have their usual meanings. For the viscosity calculations, a constant value of 3.65 Å, corresponding to the intramolecular distance Cl(t)−Cl(t), was chosen as $r_{H}$. The choice yielded good agreement with experimental viscosity data [25] for normal liquid gallium trichloride (Figure S2).

The derived viscosity for supercritical GaCl_3_ at 800 K and different pressure, shown in Figure 8c, slightly decreases as a function of pressure between 2.1 ≤ $P/P_{c}$ ≤ 3.6 and appears to be comparable with the viscosities of supercritical molecular solvents and gases [26,27,28,29]. As expected, the supercritical η(T,P) = 10^−5^ Pa s is approximately 200 times lower than the viscosity of normal gallium trichloride liquid, also plotted in Figure 8c.

## 3. Simulation Details

First-principles molecular dynamics (FPMD), implemented within the CP2K package [30], was used to investigate the local structure and atomic dynamics in molten and supercritical GaCl_3_. The generalized gradient approximation (GGA) and the PBE [31] exchange–correlation functional, along with Grimme dispersion corrections D3BJ [32], were utilized. The initial atomic configuration was generated using Empirical Potential Structure Refinement (EPSR) program [33,34]. The Lennard–Jones potential well depth ε = 1.0 kJ mol^−1^ and length σ = 2.5 Å were initially set for both gallium and chlorine. Following the preliminary equilibration, the empirical potential was introduced to start the refinement against the X-ray data until achieving internal energy stabilization. The cubic simulation box, containing 800 atoms (200 Ga and 600 Cl), was sized to match the experimental density. Subsequent optimization was conducted using density functional theory, employing the molecularly optimized correlation-consistent polarized triple-zeta valence basis set and norm-conserving relativistic Goedecker–Teter–Hutter-type pseudopotentials [35]. FPMD simulations were carried out using a canonical NVT ensemble with a Nosé–Hoover [36,37] thermostat. The simulation boxes underwent heating and cooling cycles from 300 K to 800 K using 50 or 100 K steps for duration of 30–45 ps each. The connectivity, ring statistics, and bond angle distributions were analyzed using the R. I. N. G. S. package [38] and a modified connectivity program [39].

## 4. Conclusions

First-principles simulations of supercritical gallium trichloride fluid at different pressures reveal the elementary steps of edge-sharing ES-Ga_2_Cl_6_ dimer dissociation. First, the ES-dimers convert into intermediate corner-sharing CS-Ga_2_Cl_6_ entities before further splitting into GaCl_3_ monomers. A small fraction of monomers disproportionates into molecular chlorine and gallium monochloride: GaCl_3_ → GaCl + Cl_2_. Gallium monochloride exhibits elusive behavior, forming transient species with GaCl_3_ monomers or CS-dimers via Ga-Ga homopolar or Ga-Cl heteropolar bonds (approximately 50% of the total population). The remaining 50% of the population exists as diatomic molecules.

Local tetrahedral geometry in dimers and trigonal geometry in monomers behave differently as a function of temperature and pressure. In normal liquids, the GaCl_4_ tetrahedra are strongly distorted, showing different Ga-Cl(t) (2.12 Å) and Ga-Cl(b) (2.31 Å) interatomic distances and a bimodal $B_{\mathrm{ClGaCl}}\left(\theta \right)$ bond angle distribution. Two competitive processes appear in supercritical fluids: the ES-CS conversion yields more regular tetrahedra, while temperature-induced enhanced dynamical distortion partly leads to a small fraction of non-tetrahedral geometry. Flat trigonal monomers of approximate $D_{3h}$ symmetry remain essentially intact, with symmetric and asymmetric bending vibrations temporarily decreasing the local symmetry to $C_{3v}$. A very small fraction (2.3%) of GaCl_3_ units is strongly distorted, possessing $C_{2v}$ and/or $C_{s}$ symmetry.

In contrast to normal liquids, the atomic dynamics in supercritical fluids do not exhibit the diffusion regime, i.e., the mean-square displacements $\langle r_{i}^{2}\left(t\right)\rangle \propto t^{s}$, where s = 1. Instead, the power-low exponent s appears to be intermediate between the ballistic (s = 2) and diffusion regimes, 2 < s < 1. Consequently, the diffusion coefficients calculated using the Einstein relation are only approximate. The derived viscosity η(T,P) at constant temperature (800 K) is nearly invariant over the studied pressure range (2.1 ≤ $P/P_{c}$ ≤ 3.6, where $P_{c}$ = 6.11 MPa is the critical pressure). The calculated value η(T,P) = 10^−5^ Pa s is typical for supercritical molecular solvents and gases and is 200 times lower that the viscosity of normal gallium trichloride liquid.

## Data Availability

The data presented in this study are available on request from the corresponding author.

## Supplementary Materials

The following supporting information can be downloaded at: https://www.mdpi.com/article/10.3390/molecules29061358/s1, Dissociation Reaction: Calculation Details; Figure S1: Mean-square displacements in normal liquid GaCl_3_ at 400 K; Figure S2: Experimental and FPMD-derived viscosity for molten GaCl_3_.
