# Supplementary material for: Gallium Trichloride Fluid: Dimer Dissociation Mechanism, Local Structure, and Atomic Dynamics
Source: Molecules. 2024 Mar 19;29(6):1358. doi: 10.3390/molecules29061358 (PMC10976267; doi:10.3390/molecules29061358)
Supplement: Supplementary file 1 [file molecules-29-01358-s001.zip › molecules-2910033-supplementary.pdf]

Gallium Trichloride Fluid: Dimer Dissociation Mechanism, Local Structure, and Atomic Dynamics

Maxim Khomenko [1,2], Anton Sokolov [3], Andrey Tverjanovich [4], Maria Bokova [3], Mohammad Kassem [3], Takeshi Usuki [5] and Eugene Bychkov [3,*]

[1] National Research Centre, Kurchatov Institute, Shatura, Moscow 140700, Russia; khomenkolaser@gmail.com
[2] Laboratory of Biophotonics, Tomsk State University, Tomsk 634050, Russia
[3] Laboratoire de Physico-Chimie de L'atmosphère, Université du Littoral Côte d'Opale, 59140 Dunkerque, France; anton.sokolov@univ-littoral.fr (A.S.); maria.bokova@univ-littoral.fr (M.B.); mohamad.kassem@univ-littoral.fr (M.K.)
[4] Institute of Chemistry, St. Petersburg State University, St. Petersburg 198504, Russia; andr.tver@yahoo.com
[5] Faculty of Science, Yamagata University, Yamagata 990-8560, Japan; usuki@kdw.kj.yamagata-u.ac.jp
* Correspondence: eugene.bychkov@univ-littoral.fr

SUPPORTING INFORMATION

Dissociation Reaction: Calculation Details

Figure S1. Mean-square displacements in normal liquid $GaCl_3$ at 400 K.

Figure S2. Experimental and FPMD-derived viscosity for molten $GaCl_3$.

Dissociation Reaction: Calculation Details

Let us consider the dissociation reaction:

$$Ga_2Cl_6 \rightleftharpoons 2GaCl_3 \,. \tag{S1}$$

The equilibrium constant, based on reported partial pressures[S1] (the insert in Figure 1a), can be written as

$$K_p = \frac{P^2_{GaCl_3}}{P_{Ga_2Cl_6}} \,. \tag{S2}$$

The equilibrium constant, representing the molar fractions, is given by

$$K_m = \frac{x^2_{GaCl_3}}{x_{Ga_2Cl_6}} = \frac{K_p}{P_{total}} \,. \tag{S3}$$

Solving the quadratic equation relating K_m and x_{GaCl_3} and taking into account that $x_{Ga_2Cl_6}$ + x_{GaCl_3} = 1, one obtains the monomer molar fraction x_{GaCl_3} as a function of temperature for unsaturated vapor (Figure 1b).

The equilibrium constant depends on the total pressure in the system

$$\frac{d \ln K_m}{dP} = -\frac{1}{P_{total}} \,. \tag{S4}$$

By calculating x_{GaCl_3} at P_{total} = P_c and P_{total} = $3P_c$, one observes a decreasing monomer molar fraction (Figure 1b), consistent with the experimental and FPMD results.

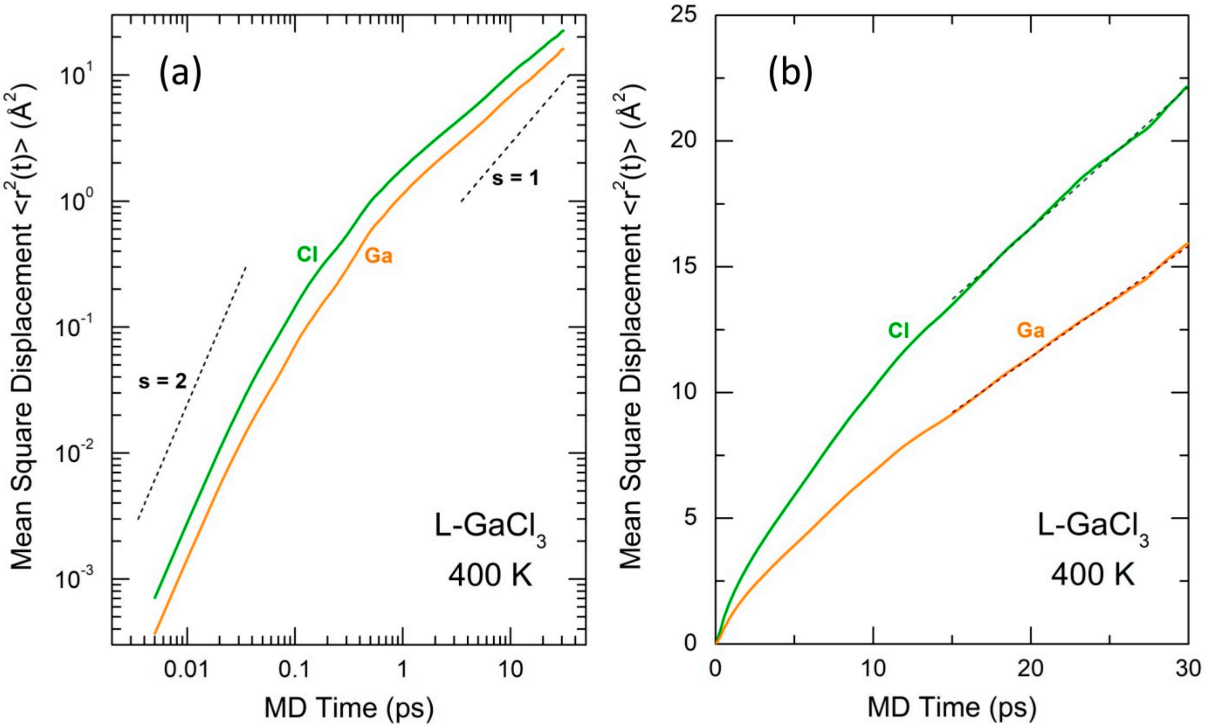

Figure S1. Mean-square displacements in normal liquid $GaCl_3$ at 400 K: (a) log-log scale, (b) linear scale. The slopes for ballistic regime (s = 2) and diffusion regime (s = 1) are shown in (a).

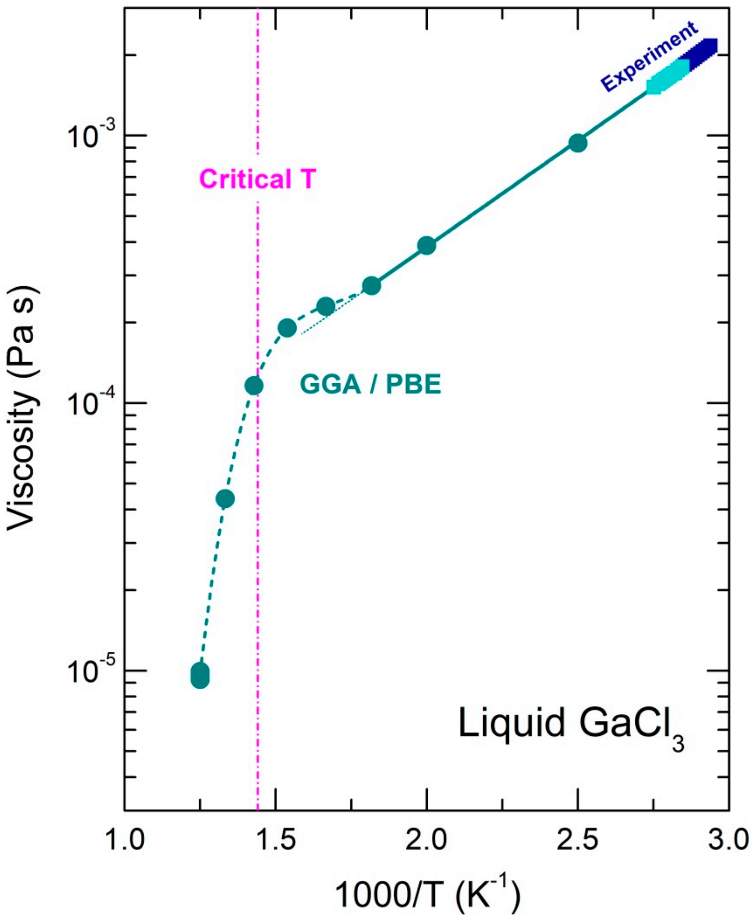

Figure S2. Experimental[S2] and FPMD-derived viscosity for molten $GaCl_3$.

Additional References

S1. Fischer, W.; Jübermann, O. Über thermische Eigenschaften von Halogeniden. 10. Dampfdrucke und Dampfdichten von Gallium III – Halogeniden. *Z. Anorg. Allg. Chem.* **1936**, *227*, 227–236.

S2. Greenwood, N. N.; Wade, K. Some Physical Properties of Molten and Supercooled Gallium Trichloride. *J. Inorg. Nucl. Chem.* **1957**, *3*, 349–356.
